# Cellulose and Its Derivatives-Based Skin Dressings: Design, Smart Advances and Applications

**DOI:** 10.3390/pharmaceutics18050562

**Published:** 2026-04-30

**Authors:** Shiyan Wang, Yu Wang, Mengran Guo

**Affiliations:** Department of Pharmacy, West China Hospital, Sichuan University, Chengdu 610041, China; wang_sy95@scu.edu.cn (S.W.); yuu1999@163.com (Y.W.)

**Keywords:** cellulose and its derivatives, skin dressings, smart responsive systems, wound healing, bacterial infections, biomimetic scaffolds, drug delivery

## Abstract

The treatment of skin diseases remains a significant clinical challenge. Cellulose and its derivatives have emerged as research hotspots in skin-related applications due to their excellent biocompatibility, structural modifiability, and biomimetic properties. This review systematically summarizes the diverse construction forms of cellulose-based materials, including films, nanofibrous scaffolds, hydrogels, and aerogels, with a focus on smart responsive systems tailored to various microenvironmental conditions. Their application progresses in acute/chronic wound healing, bacterial infections, burns, scar prevention, immunomodulation, and smart wearable monitoring are highlighted. The underlying mechanisms involving anti-infection, pro-regeneration, microenvironment modulation, and sensing are analyzed, aiming to provide insights for further exploration of cellulose-based materials in skin disease therapy and even smart wearable devices.

## 1. Introduction

In various skin disorders, conditions represented by chronic wounds and drug-resistant bacterial infections are particularly challenging to treat [1,2,3]. Trauma to the skin surface often disrupts the local immune microenvironment homeostasis, thereby rendering the skin highly susceptible to polymicrobial infections. The refractory nature of chronic wounds is characterized by a sustained arrest in the healing process, manifested as recalcitrant bacterial biofilms, persistent inflammatory responses coupled with oxidative stress imbalance, and progressive inactivation of cellular functions [4,5]. Concurrently, the treatment of drug-resistant infections is impeded by the natural barrier function of the stratum corneum, resulting in poor local drug penetration and difficulty in achieving effective therapeutic concentrations deep within the lesion [6]. Furthermore, conventional topical formulations commonly suffer from poor retention and uncontrolled release in the exudate-rich wound microenvironment and lack the capacity to actively modulate the wound milieu to induce tissue regeneration [7]. Therefore, the development of novel dressings systems capable of efficiently penetrating physiological barriers, achieving intelligent drug release, exhibiting anti-infective activity, and supporting tissue repair has emerged as a critical scientific challenge urgently needing resolution in the current landscape of skin disease therapy [8].

In the design of skin dressings, the selection of materials is a pivotal factor determining their therapeutic efficacy and application prospects. Cellulose and its derivatives have become exceptionally promising carrier materials in this field, owing to their distinct advantages of wide availability, excellent biocompatibility, and inherent potential for structural engineering modification [9]. Cellulose, as the most abundant natural polymer in nature, inherently possesses favorable biodegradability and mechanical properties [10,11]. However, the core factor driving its widespread application in the biomedical field lies in its diverse functionalized forms. Derivatives obtained through chemical modification of cellulose, such as carboxymethyl cellulose (CMC), exhibit superior water solubility, film-forming capacity, and high water-holding capacity due to the introduction of hydrophilic carboxymethyl groups. These properties enable effective absorption of wound exudate and provide an ideal moist environment conducive to wound healing [12,13]. Furthermore, nanocellulose, isolated from natural cellulose via physical or chemical methods, exhibits unique nanoscale effects. Based on their morphology and structural characteristics, nanocellulose is primarily classified into three types: cellulose nanocrystals (CNC), characterized by high crystallinity and a rod-like morphology; cellulose nanofibrils (CNF), containing amorphous regions and presenting a flexible network structure; and bacterial cellulose (BC), synthesized by microorganisms, which naturally forms a high-purity three-dimensional nanofiber network [14,15,16,17,18,19,20]. These three types of nanocellulose all possess an exceptionally high specific surface area, outstanding mechanical strength, and closely mimic the nanofibrous topology of the native extracellular matrix, thereby conferring unique biological advantages for their use as skin drug delivery vehicles [21]. Specifically, their advantages are first manifested in superior water-holding capacity; notably, BC can bind water at levels exceeding one hundred times its own dry weight, perfectly aligning with the principles of modern moist wound therapy [22]. Secondly, their nanoscale fibrous network effectively simulates the physical architecture of the extracellular matrix, providing a biomimetic scaffold for the adhesion, migration, and proliferation of cells such as fibroblasts and keratinocytes, thereby directly participating in and promoting tissue regeneration processes [23,24,25,26]. Critically, their high degree of modifiability is paramount: the abundant reactive hydroxyl groups along the cellulose chain offer ample sites for loading therapeutics such as antibiotics, growth factors, antimicrobial nanoparticles (e.g., silver nanoparticles), or other bioactive molecules via chemical grafting or physical adsorption. Concurrently, they can be combined with other functional materials (e.g., chitosan, nanoclays) to precisely tune drug release kinetics, thereby achieving a transformative leap from passive drug release to intelligently responsive delivery [27,28,29,30]. Based on these characteristics, this review searched PubMed using the query (“cellulose” OR “cellulose derivatives”) AND (“dressings” OR “films” OR “nanofibrous scaffolds” OR “hydrogels” OR “aerogels”) AND “skin” to systematically summarize the representative research of cellulose and its derivatives-based dressings published from 2020 to 2026, and to delineate the research progress of different types of cellulosic materials in the field of skin drug delivery, with the goal of providing a theoretical reference and empirical insights for the future in-depth application of cellulosic materials in the treatment of skin diseases.

## 2. Construction Forms of Cellulose and Its Derivatives-Based Dressings

**Figure 1:**
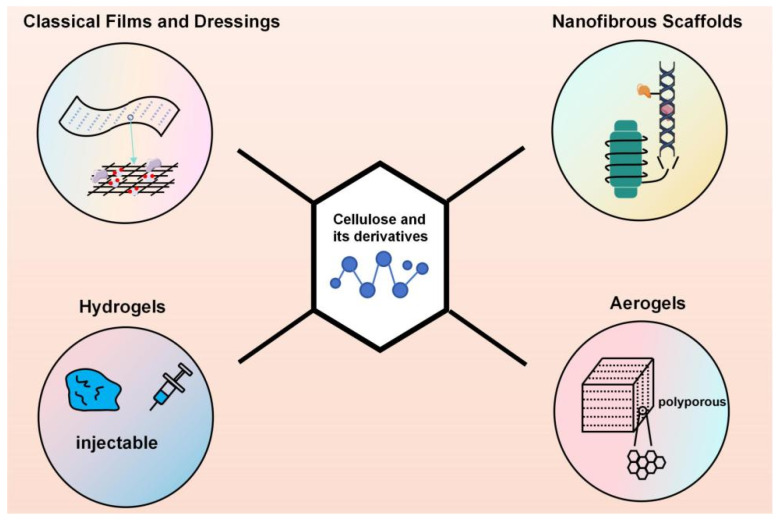
Construction forms of cellulose and its derivatives-based dressings.

**Figure 2:**
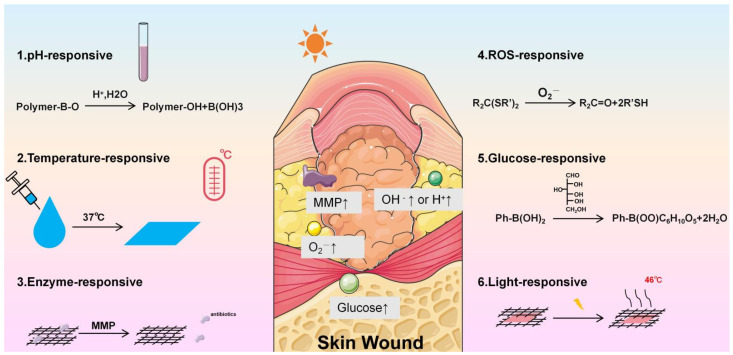
Smart responsive systems of cellulose and its derivatives-based dressings.

Cellulose and its derivatives-based materials can be processed into various dosage forms tailored to meet diverse clinical requirements through different physical or chemical methods (shown in Figure 1 and Figure 2). Their morphologies encompass a wide range of structures, from two-dimensional films to three-dimensional porous scaffolds, each with distinct characteristics. The common goal of these forms is to achieve a synergistic enhancement between the therapeutic efficacy of the drug and the inherent biological functions of the material itself.

### 2.1. Classical Films and Dressings

Films and dressings represent the most intuitive and widely used conventional dosage forms for topical drug administration. Dressings based on cellulose, particularly BC, signify a modern innovation of this classic form. Unlike traditional textile or non-woven fabric dressings, BC dressings consist of a natural nanofiber network synthesized by microorganisms (e.g., *Komagataeibacter xylinus*) through static or dynamic fermentation [31,32]. This unique biosynthesis process endows them with a series of exceptional properties. Firstly, BC possesses extremely high purity and biocompatibility, as it is free from plant-derived impurities like lignin and pectin, thereby eliciting minimal immune rejection [33,34]. Secondly, its inherent three-dimensional nanofiber network (with fiber diameters of approximately 20–100 nm) exhibits remarkable water-holding capacity, capable of binding water at levels exceeding one hundred times its own dry weight. This not only enables effective absorption of wound exudate but also creates an ideal moist microenvironment at the wound site by locking in moisture [35,36]. Furthermore, BC maintains excellent mechanical strength and flexibility in its hydrated state, allowing it to conform tightly to irregular wound surfaces and function as an effective physical barrier [37,38].

In recent years, research has moved beyond the native form of BC, achieving functionalization through in situ compositing or post-treatment loading techniques. For instance, antimicrobial agents (e.g., chitosan), analgesics, or growth factors can be directly added to the fermentation medium, becoming integrated during the formation of the BC network [39,40,41,42]. A recent innovative study compounded Laponite RD clay nanoplatelets with BC to fabricate functional films. The incorporation of clay not only enhanced the mechanical properties of the film, but its layered structure also enabled efficient loading of antibiotics (e.g., ciprofloxacin). By leveraging ion-exchange mechanisms, precise control over drug release kinetics was achieved, effectively preventing the initial burst release commonly associated with conventional drug-loaded dressings. This resulted in long-lasting, controllable antibacterial therapy [43]. This research clearly demonstrates the potential of traditional film forms to evolve into intelligent controlled-release systems through nanocomposite technology.

### 2.2. Nanofibrous Scaffolds

Nanofibrous scaffolds, prepared via physical techniques such as electrospinning, represent one of the most actively researched dressings forms currently [44,45,46]. Their core advantage lies in the ability to construct two- or three-dimensional fibrous networks characterized by an exceptionally high specific surface area and a highly interconnected porous structure [47]. From a biological perspective, this architecture closely mimics the nanofibrous topography of the native extracellular matrix, providing an ideal microenvironment for cell adhesion, migration, and proliferation [48,49]. From a pharmaceutical standpoint, the immense specific surface area offers ample space for drug loading, while the porous structure facilitates cell infiltration, nutrient exchange, and metabolic waste removal [50,51].

Cellulose derivatives with good spinnability, such as sodium CMC or cellulose acetate (CA), are commonly chosen for fabricating such scaffolds [52,53]. These materials can be electrospun alone but are often blended with other polymers possessing excellent fiber-forming capabilities (e.g., polyvinyl alcohol PVA, polyethylene oxide PEO, or chitosan) to optimize the spinning process and the mechanical properties of the resulting fiber membrane [54,55]. The methods for drug loading are versatile: drugs can be directly incorporated into the spinning precursor solution for blend electrospinning, achieving uniform distribution within the fibers; alternatively, drugs can be loaded post-fiber formation via physical adsorption or chemical grafting [56,57,58]. The study by Seyedi et al. serves as a classic example of this approach. They fabricated CMC-based nanofiber mats loaded simultaneously with silver nanoparticles (AgNPs) and the antioxidant resveratrol via electrospinning. The AgNPs provided potent, broad-spectrum antibacterial activity, while resveratrol mitigated oxidative stress and suppressed inflammatory responses by scavenging excessive reactive oxygen species (ROS). This synergistic combination significantly promoted the regenerative healing of infected skin wounds in animal models [59]. This research highlights the unique advantages of nanofibrous scaffolds as multifunctional, synergistic therapeutic platforms.

### 2.3. Hydrogels

Hydrogels are three-dimensional hydrophilic polymer networks formed through physical or chemical crosslinking, capable of absorbing and retaining large amounts of water without dissolving [60]. Cellulose-based hydrogels have emerged as ideal materials in the wound care field due to their perfect alignment with the modern concept of “moist wound healing” [61]. The abundant hydroxyl groups on the cellulose molecular chain facilitate the formation of stable gel networks through physical entanglement, hydrogen bonding, or reactions with crosslinking agents (e.g., glutaraldehyde, metal ions) [62]. In skin drug delivery systems, cellulose-based hydrogels play a dual role: first, as drug reservoirs, their hydrated networks can encapsulate both hydrophilic and hydrophobic drug molecules, enabling sustained drug release via diffusion or network degradation [63]. Secondly, as wound microenvironment modulators, they not only maintain wound moisture and alleviate pain but also control inflammatory responses by absorbing excess exudate and provide a soft matrix support for epithelial cell migration [64]. In recent years, research frontiers have focused on endowing hydrogels with injectability and self-healing properties. For instance, combining oxidized CNF (TOCNF) with dynamic crosslinkers (e.g., borax) can yield injectable hydrogels with shear-thinning behavior. These hydrogels can fill irregularly shaped wound cavities and rapidly revert to a gel state in vivo, achieving minimally invasive administration and perfect wound conformity [65].

Furthermore, “bioinks” based on cellulose or its derivatives (e.g., nanocellulose, CMC) are critical materials for 3D bioprinting of soft tissue scaffolds [66]. Such bioinks must possess appropriate rheological properties (e.g., shear-thinning behavior, rapid modulus recovery) to ensure extrudability during printing and shape fidelity post-printing [67]. Currently, researchers are dedicated to developing cellulose-based bioinks loaded with living cells (e.g., fibroblasts, keratinocytes) and growth factors, aiming to print bioactive, personalized skin substitutes or wound dressings [68,69].

### 2.4. Aerogels

Aerogels are porous solid materials obtained by replacing the liquid phase within a hydrogel or alcogel with a gas (typically air). They retain the original three-dimensional network structure of the gel, resulting in an extremely high porosity (>90%), ultra-low density, and a massive specific surface area [70,71,72]. Depending on the preparation process, aerogels can be fabricated using techniques such as supercritical CO_2_ drying, freeze-drying, or ambient pressure drying. Among these, supercritical CO_2_ drying is widely employed for its ability to best preserve the integrity of the network structure [73,74]. Nanocellulose, particularly CNF and CNC, has become an ideal “building block” for constructing bio-based aerogels, owing to its nanoscale effects and abundant surface hydroxyl groups [75]. Studies indicate that cellulose-based aerogels can achieve densities as low as 0.01–0.25 g/cm^3^, specific surface areas ranging from 100 to 300 m^2^/g, and porosity typically exceeding 85%, with some materials reaching over 98% [76]. For example, Rostamitabar et al. prepared cellulose-chitosan composite aerogel microfibers via wet spinning combined with supercritical CO_2_ drying. These microfibers exhibited a low density of approximately 0.18 g/cm^3^, high porosity of about 85%, and a large specific surface area of roughly 300 m^2^/g. Their unique “macroporous shell-nanoporous core” structure provides an ideal space for drug loading [74].

In the field of skin drug delivery systems, the core advantages of cellulose aerogels lie in their exceptional drug loading capacity and unique pore structure. Their interconnected hierarchical channels, ranging from nano- to micrometers, can efficiently adsorb and accommodate drugs, proteins (e.g., growth factors), and even nanoparticles in amounts far exceeding their own mass [77,78]. Li et al. reported that by adjusting the ratio of CNF to gelatin and the degree of crosslinking, the loading efficiency for 5-fluorouracil could be increased from 10% to 35%. Li et al. prepared carboxylated cellulose composite aerogels achieving a 5-fluorouracil loading capacity of up to 180 mg/g [75]. Regarding drug release behavior, cellulose aerogels can achieve sustained release lasting from several hours to several days. For instance, ibuprofen-loaded cellulose-chitosan aerogel microfibers demonstrated continuous drug release over 48 h [74]; similarly, ibuprofen-loaded chitosan/silk fibroin nanofibrous aerogels exhibited controlled drug delivery performance and an exudate absorption rate exceeding 100% [73]. The “sponge-like” structure offers novel strategies for wound exudate management: on the one hand, cellulose aerogels can rapidly absorb large volumes of wound exudate, with absorption rates reaching over 400% of their own weight, effectively preventing wound maceration; on the other hand, through “smart” drainage, they maintain an optimally moist wound environment conducive to cell migration and tissue regeneration [77,79]. As research progresses, functional cellulose aerogels are evolving towards smart-responsive and multifunctional integrated systems. By incorporating pH-responsive polymers (e.g., polyacrylic acid), thermo-sensitive materials (e.g., poly(N-isopropylacrylamide)), or enzyme-sensitive crosslinking bonds, dynamic responses to changes in the wound microenvironment and on-demand drug release can be achieved. Concurrently, loading antibacterial nanoparticles (e.g., AgNPs), anti-inflammatory drugs, and pro-angiogenic factors within a single aerogel system enables the construction of multifunctional delivery platforms integrating anti-infection, anti-inflammatory, and pro-regeneration capabilities, offering comprehensive solutions for the treatment of complex wounds [80,81,82,83].

### 2.5. Smart Responsive Systems

With the advancement of materials science and the deepening understanding of wound pathophysiology, smart responsive systems capable of sensing and dynamically responding to microenvironmental changes have become a crucial development direction for cellulose-based drug delivery systems [84]. The design concept of these materials leverages pathological signals unique to the chronic wound or infected microenvironment—such as acidic pH, elevated reactive oxygen species (ROS) levels, specific enzyme activity, or high glucose concentrations—as “triggers.” This enables on-demand drug release or autonomous regulation of material functions, thereby overcoming the limitations of traditional dressings that rely on “passive release” and achieving precision therapy [85,86]. Based on the type of triggering signal, cellulose-based smart responsive systems can be primarily classified into the following categories, with representative studies summarized in Table 1.

#### 2.5.1. pH-Responsive Systems

pH responsiveness is the most extensively studied type of smart response, with its design rationale rooted in the classic pH dynamics of the wound microenvironment: normal skin is weakly acidic (pH 5.0–6.0), while acute wounds initially exhibit a neutral or weakly alkaline pH. Chronically infected wounds, however, can become acidic again (pH 5.0–6.5) due to bacterial metabolic activity producing acids [106,107,108,109]. Cellulose-based pH-responsive gels are primarily achieved by introducing ionizable groups or dynamic covalent bonds, such as Schiff base bonds (-C=N-) [110,111]. For instance, ionizable boric acid readily forms reversible covalent bonds with alginate (which contains 1,2-diol groups), leading to a more hydrophilic structure. When the hydrogel is exposed to the acidic environment of a wound, the boronate ester bonds (-B-O-) dissociate, causing disruption of the hydrogel structure and consequently promoting drug release [110]. Yang et al. prepared a Schiff base hydrogel based on chitosan and dialdehyde carboxymethyl cellulose (DA-CMC). This system exhibited the highest swelling ratio and drug release at pH 7.0, and in vivo studies demonstrated a wound healing rate exceeding 95% after 11 days of treatment [90]. Furthermore, pH responsiveness can also be achieved by incorporating pH-sensitive polymers or nanoparticles into the gel, or through acid-catalyzed hydrolysis and electrostatic interactions [112,113,114,115,116].

#### 2.5.2. Temperature-Responsive Systems

Temperature-responsive systems can undergo swelling-shrinking or sol–gel phase transitions near a specific temperature (typically body temperature), a property that makes them suitable as injectable drug delivery vehicles [117,118]. Poly(N-isopropylacrylamide) (PNIPAM) is the most commonly used thermosensitive polymer due to its lower critical solution temperature (LCST) of approximately 32 °C, which is close to body temperature. Research shows that incorporating CNC into a PNIPAM network significantly enhances the mechanical properties of the hydrogel. This composite system exhibits a volume phase transition temperature (VPTT) of 36–39 °C and displays a “burst-sustained release” biphasic release profile at 37 °C, positioning it as a promising injectable wound dressing [119].

#### 2.5.3. Enzyme-Responsive Systems

The design of enzyme-responsive systems is based on the pathophysiological characteristic of significantly elevated activity of specific enzymes, such as matrix metalloproteinases (MMPs), in the chronic wound microenvironment [120,121,122]. By incorporating peptide sequences or chemical bonds that can be specifically recognized and cleaved by these enzymes into cellulose-based materials, targeted drug release can be achieved in regions with high enzyme activity [123,124]. For example, the research group led by Senerovic utilized bacterial nanocellulose as an immobilization matrix for the lactonase enzyme YtnP. This achieved long-term enzyme stability (retaining activity for 3 months at 4 °C) and significantly accelerated healing while reducing inflammation in an animal wound model infected with Pseudomonas aeruginosa. This work provides a foundation for developing smart wound dressings based on enzyme immobilization technology [125].

#### 2.5.4. Reactive Oxygen Species (ROS)-Responsive Systems

Chronic wounds, particularly diabetic ulcers, are often characterized by persistently elevated oxidative stress levels, where excessive ROS is a key factor leading to healing arrest [126,127,128]. ROS-responsive systems incorporate chemical structures specifically cleavable by ROS, such as oxalate esters (R–O–C(=O)–C(=O)–O–R′) or thioketals (R_2_C(SR′)_2_). This enables drug release within the high-ROS microenvironment or confers the ability to scavenge ROS themselves [129,130,131]. For instance, Rong et al. developed an antioxidant nanodrug based on cysteamine-modified cellulose nanospheres (Cys-Cel NS) and combined it with a thermosensitive gel for diabetic wound treatment. In vivo experiments confirmed that this gel reduced ROS levels, modulated the oxidative microenvironment of diabetic wounds, and led to significant wound healing after 13 days of treatment [97].

#### 2.5.5. Glucose-Responsive Systems

Glucose-responsive systems are primarily designed for chronic wounds in diabetic patients. They utilize the glucose-recognizing ability of glucose oxidase (Gox) or phenylboronic acid (PBA) and its derivatives to achieve drug release under hyperglycemic conditions [132,133]. Previous research successfully prepared a cellulose/4-vinylphenylboronic acid (VPBA) composite hydrogel via electron beam irradiation. The incorporation of PBA groups endowed the system with dual pH and glucose responsiveness, demonstrating self-regulating insulin release behavior in glucose solutions of varying concentrations [134]. A microneedle system crosslinked with CNC and PBA achieved glucose concentration-dependent drug release, offering a novel platform for non-invasive transdermal drug delivery [135]. Furthermore, Chen et al. employed metabolic glycoengineering combined with click chemistry to covalently immobilize Gox onto BC, constructing a cascade catalytic dressing capable of simultaneously reducing local glucose levels and scavenging ROS. This dressing significantly accelerated wound healing in a diabetic mouse model [136].

#### 2.5.6. Light-Responsive Systems

Light-responsive systems incorporate photothermal agents, such as polydopamine, carbon quantum dots, or MXene, to generate heat or ROS upon exposure to specific wavelengths of light, enabling photothermal or photodynamic therapy [137,138,139]. In recent years, various cellulose-based light-responsive hydrogels have been successfully developed. For example, Xie et al. introduced MXene nanosheets into a water-soluble cellulose network, creating a multifunctional hydrogel with both photothermal conversion and electrical conductivity. Under solar irradiation, the surface temperature of this hydrogel reached 100 °C [140]. Chen et al. designed a pH-induced, self-enhancing photothermal cellulose nanocrystal hydrogel. Utilizing polyaniline-modified CNC, it achieved precise photothermal antibacterial therapy at 45–48 °C within the acidic microenvironment of infected wounds, effectively avoiding overheating damage to surrounding healthy tissue [141]. Additionally, oxidized CMC hydrogels loaded with biosynthesized CuS nanoparticles and functionalized MoS_2_/cellulose non-woven composites have also demonstrated excellent photothermal antibacterial properties, providing new strategies for treating infected wounds caused by drug-resistant bacteria [142,143].

#### 2.5.7. Multi-Responsive Systems

Integrating two or more response mechanisms into a single drug delivery system enables more precise regulation of drug release, offering intelligent solutions tailored to the complex pathological features of the wound microenvironment. Targeting the multifaceted challenges of diabetic wounds—including hyperglycemia, severe oxidative stress, persistent bacterial infection, and complex pH fluctuations—Hua et al. developed an injectable multifunctional hydrogel (QPTx) based on dynamic Schiff base bonds and phenylboronate esters [105]. This hydrogel was constructed via dynamic covalent crosslinking among phenylboronic acid-modified quaternized chitosan (QCS-PBA), polydopamine-coated tunicate CNC (PDA@TCNCs), and polyvinyl alcohol (PVA), achieving triple responsiveness to pH, temperature, and glucose. The system can load insulin for on-demand release in response to the complex and variable diabetic wound environment. Furthermore, the incorporation of PDA@TCNCs endowed the hydrogel with photothermal antibacterial activity, enhanced adhesiveness, and antioxidant properties. In a diabetic full-thickness skin defect model, this hydrogel dressing significantly promoted wound healing, outperforming the commercial dressing Tegaderm™.

## 3. Applications in Various Skin Conditions

**Figure 3:**
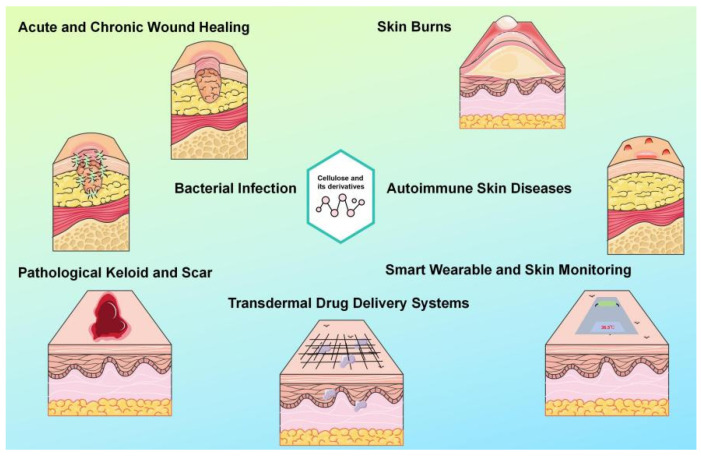
Applications of cellulose and its derivatives-based dressings in various skin conditions.

Building upon the diverse construction forms and advanced functionalization techniques discussed previously, cellulose-based dressings systems have progressively expanded from fundamental research towards application-oriented exploration for specific skin diseases, demonstrating significant potential for clinical translation (shown in Figure 3).

### 3.1. Acute and Chronic Wound Healing

Wound healing is a highly coordinated and dynamic biological process involving four interrelated phases: hemostasis, inflammation, proliferation, and remodeling [144,145]. The fundamental distinction between acute and chronic wounds lies in the arrest of the healing process in the latter, typically stalled in the inflammatory or proliferative phase, resulting in a pathological state that is difficult to heal spontaneously [146,147]. Through multifunctional integrated design, cellulose-based systems aim to disrupt this stagnant state and synergistically advance the various stages of the healing process [148].

#### 3.1.1. Hemostasis and Coagulation

The initial phase of wound healing is the hemostatic phase, where rapid and effective hemostasis is crucial for preventing blood loss and initiating subsequent repair processes [149]. Traditional dressings primarily rely on physical compression to achieve hemostasis, whereas cellulose-based materials, leveraging their unique porous structure and surface chemical properties, can actively participate in the coagulation process through multiple mechanisms [150]. Research indicates that functional modification of cellulose can significantly enhance its hemostatic performance [151]. Kamlesh et al. developed a sponge-like hemostatic dressing (HBC-PDA) based on bacterial cellulose (BC) coated with polydopamine (PDA) nanospheres. This material exhibited rapid fluid absorption capacity and excellent coagulation activity, achieving 90% clotting efficiency within 90 s, with the amine groups in PDA playing a key role in activating the intrinsic coagulation pathway [152]. Ma et al. designed and prepared a porous SiO_2_/ZnO-carboxymethyl cellulose (CMC) composite hydrogel. Its unique micro-nano porous structure promoted erythrocyte adhesion and activation; combined with the synergistic effect of SiO_2_ and ZnO, the clotting time was significantly shortened to 98 ± 18 s, while also endowing the material with excellent antibacterial properties [153]. Furthermore, Chen et al. developed a photoelectric-coupled Mxene–bacterial cellulose sponge dressing. The Mxene modification not only enhanced conductivity, enabling active coupling with the endogenous electric field to accelerate healing, but also demonstrated excellent photothermal antibacterial activity (98% antibacterial rate) and rapid hemostatic performance [154]. Mai et al. reported a bacteria-responsive cellulose-based fabric dressing (Fabric-PAC). Modified with polyphosphate (PolyP), this dressing exposes PolyP upon contact with bacteria, triggering coagulation and platelet-driven tissue remodeling, thereby achieving on-demand synergy between antibacterial and hemostatic functions [155]. These studies demonstrate that combining the porous structure of cellulose-based materials with functional components or intelligent response mechanisms can significantly enhance their hemostatic performance, providing new strategies for developing multifunctional dressings that integrate rapid hemostasis and pro-healing capabilities.

#### 3.1.2. Anti-Infection and Anti-Inflammation

Infection and persistent inflammation are primary obstacles hindering chronic wound healing [156,157]. Cellulose-based materials establish an active defense system by combining physical barrier function with the loading and local sustained release of chemical antimicrobial agents [80,158,159]. For instance, one study prepared resveratrol (RSV)-loaded cellulose acetate butyrate (CAB) nanoparticles via the solvent evaporation method. These were subsequently integrated into lyophilized wafers primarily composed of sodium CMC compounded with hydroxypropyl methylcellulose (HPMC). This design aimed to encapsulate RSV within CAB nanoparticles to improve its solubility and stability, while leveraging the porous, highly absorbent structure of the CMC/HPMC composite wafers to provide a moist environment for the wound. In vitro studies showed that the optimized RSV nanoparticles had a mean particle size of approximately 248.5 nm and an encapsulation efficiency of 87.58%, enabling sustained release from the wafers. Resveratrol can mitigate oxidative stress damage by scavenging excessive reactive oxygen species (ROS) at the wound site and downregulate the expression of pro-inflammatory factors. In a rat full-thickness skin defect model, these drug-loaded wafers significantly accelerated wound closure, confirming their efficacy as a platform for delivering RSV to promote wound healing [160]. In the aforementioned study on the functional film fabricated by compounding BC with Laponite RD nanoclay for the controlled release of ciprofloxacin, the clay nanoplatelets adsorbed and slowly released the antibiotic via an ion-exchange mechanism. This achieved smooth release over several days, effectively avoiding the initial “burst release” problem common in traditional drug-loaded dressings, thus offering an ideal solution for long-term anti-infective therapy [43].

#### 3.1.3. Promoting Cell Proliferation and Tissue Regeneration

Following the control of infection and inflammation, initiating and accelerating tissue regeneration is a critical step in wound healing [161,162]. Cellulose-based materials exhibit unique advantages in this regard. On the one hand, the inherent three-dimensional nanofibrous network of nanocellulose (e.g., CNF, BC) structurally mimics the native extracellular matrix (ECM), providing an ideal physical scaffold for the adhesion, migration, and proliferation of fibroblasts, keratinocytes, and endothelial cells [18,163,164,165]. Studies have shown that surface charge modification of CNF—for example, coating with positively charged polylysine or negatively charged heparin—can specifically guide the behavior of different cell types, such as mesenchymal stem cells and fibroblasts, thereby directionally promoting granulation tissue formation or re-epithelialization [166,167]. On the other hand, cellulosic materials serve as efficient “reservoirs” and “delivery vehicles” for bioactive molecules. Loading and controlled release of growth factors like vascular endothelial growth factor (VEGF), epidermal growth factor (EGF), and basic fibroblast growth factor (bFGF) can directly stimulate angiogenesis and cell proliferation [168,169,170,171,172]. For example, combining VEGF with a thermosensitive cellulose derivative hydrogel enables in situ gelation at wound temperature and sustained VEGF release, effectively promoting wound vascularization [173].

#### 3.1.4. Modulating the Wound Microenvironment

Ideal wound dressings should possess the ability to dynamically regulate the wound microenvironment. Cellulose and its derivatives, with their inherent high hydrophilicity and porous structure, are excellent “microenvironment managers.” Dressings or hydrogels made from cellulose derivatives like CMC or alginate exhibit exceptionally high fluid absorption capacity, effectively taking up excess wound exudate, reducing maceration, and maintaining an appropriately moist environment at the dressing–wound interface through water retention [174,175,176]. Furthermore, although BC dressings swell upon fluid absorption, their nanofibrous network structure retains excellent gas permeability, allowing oxygen exchange and inhibiting anaerobic bacterial growth, while effectively blocking external bacterial invasion [177].

### 3.2. Bacterial Infectious Skin Diseases

For localized bacterial infectious skin diseases such as impetigo, folliculitis, furuncles, and carbuncles, the core of treatment lies in maintaining drug levels above the minimum inhibitory concentration (MIC) at the infection site [178,179]. The advantage of topical administration is the ability to achieve high and sustained drug concentrations directly at the infected site, thereby enhancing bactericidal efficacy and reducing systemic side effects [180]. Traditional topical ointments suffer from limitations such as short residence time, limited permeability, and the need for frequent application, which restrict their clinical effectiveness [181]. Cellulose-based drug delivery systems offer novel strategies to address these challenges. Their core strategy involves constructing sustained-release platforms for topical antibiotics or antimicrobial agents. By loading antibiotics (e.g., tetracycline, ciprofloxacin) or antimicrobial agents (e.g., silver ions) into cellulose-based films, hydrogels, or nanofibers, the drugs are slowly released from the material via diffusion, matrix degradation, or environmentally responsive mechanisms [182,183,184,185,186,187]. This not only maintains an effective therapeutic concentration at the infection site for a prolonged period, enhancing bactericidal efficacy, but also significantly reduces systemic side effects and dosing frequency caused by systemic absorption or rapid clearance, thereby improving patient compliance [188,189]. Facing the increasingly severe challenge of antibiotic resistance, cellulose-based materials provide ideal carriers for combination therapies and novel antimicrobial strategies. For instance, co-loading antibiotics with antimicrobial peptides (AMPs) or nitric oxide (NO) donors can generate synergistic antibacterial effects and reduce the risk of bacterial resistance. Cellulose dressings containing photothermal agents (e.g., polydopamine, carbon quantum dots) can generate localized heat upon near-infrared irradiation, achieving physical bacterial eradication, particularly effective against drug-resistant strains [190,191,192].

### 3.3. Skin Burns

The treatment of burn wounds faces multiple challenges, including severe pain, massive fluid and protein loss, high infection risk, and difficulty in dermal regeneration following deep burns [193,194,195]. The value of cellulose-based materials in this area is primarily manifested in the following aspects: firstly, they serve as ideal bioactive barriers. High-purity BC dressings can be applied directly to second-degree burn wounds; their moist, flexible nature can significantly alleviate pain, and their semi-permeability prevents excessive fluid evaporation and maintains electrolyte balance while allowing the drainage of some toxins from the exudate [95,196,197]. Secondly, their potent drug delivery capacity is utilized by loading and releasing analgesics (e.g., lidocaine) and antimicrobial/anti-inflammatory agents (e.g., silver sulfadiazine, zinc oxide, curcumin) for managing infection and pain in the early stages of burns [198,199,200,201,202,203]. Furthermore, a water-soluble CMC sodium/sodium alginate/chitosan (CMC-Na/SA/CS) composite hydrogel, through its self-regulating and anti-adhesive properties, upregulates VEGF expression and downregulates bFGF expression in the early phase of burn healing, while inducing bFGF upregulation in the later phase. Simultaneously, this dressing reduces wound levels of tumor necrosis factor-alpha (TNF-α) and interleukin-6 (IL-6), thereby promoting burn healing [204]. For deep burns, 3D-printed cellulose composite scaffolds show immense potential. For example, research utilizing a nanocellulose/gelatin methacryloyl (GelMA) composite bioink integrated with carbon quantum dots has enabled the printing of bilayer scaffolds with biomimetic structures. These scaffolds not only fill tissue defects, but the carbon quantum dots also confer multifunctional synergistic effects including photothermal antibacterial activity, anti-inflammation, and promotion of cell migration, offering an integrated solution combining anti-infection and pro-regeneration capabilities for repairing deep burns complicated by severe infection [205,206,207].

### 3.4. Applications in Other Conditions

With the progression of fundamental research, the application of cellulose-based dressings is expanding into a broader spectrum of skin conditions.

#### 3.4.1. Pathological Keloid and Scar

The formation of hypertrophic scars and keloids is associated with excessive fibroblast proliferation and aberrant collagen deposition during wound healing. Cellulose-based hydrogels or films can serve as controlled-release carriers for anti-scarring drugs, enabling targeted intervention in scar formation. Li et al. developed a cellulose nanofiber scaffold loaded with sitagliptin (SITA) and zinc sulfide nanoparticles (NZnS), designated CNF@SITA@NZnS, designed to inhibit DPP4-positive fibroblasts for regenerative healing [208]. This scaffold significantly promoted scarless healing and hair follicle regeneration in rat models, with in vivo experiments demonstrating reduced skin fibrosis, improved collagen ratio, and increased formation of new hair follicles at the wound site. In vitro studies revealed that the scaffold material promoted scarless healing, possibly by inhibiting extracellular matrix secretion and fibroblast-to-myofibroblast conversion. RNA sequencing elucidated the underlying mechanisms, suggesting activation of the ECM-receptor interaction pathway favorable to the wound healing process. Zikmundová et al. loaded bacterial nanocellulose with curcumin and its thermal degradation products, investigating their effects on human dermal fibroblasts, and found that nanocellulose loaded with pure curcumin exhibited good biocompatibility with fibroblasts while demonstrating enhanced antibacterial activity [209]. A more promising strategy involves designing responsive systems, such as cellulose-based hydrogels sensitive to matrix metalloproteinases (MMPs) which are highly expressed in scar tissue, enabling degradation and drug release upon MMP exposure for more precise targeted therapy—a direction warranting further exploration [210].

#### 3.4.2. Autoimmune Skin Diseases

Local immunomodulation is crucial in the treatment of immuno-inflammatory skin diseases like atopic dermatitis and psoriasis. Cellulose-based microparticles or nanofibers can act as delivery systems for immunosuppressants (e.g., tacrolimus) or biologics (e.g., cytokine inhibitors) [211]. Their sustained-release properties help maintain effective drug concentrations locally in the lesion, improving efficacy and reducing the risk of systemic immunosuppression [212]. Furthermore, nanocellulose itself has been found to possess certain immunomodulatory potential; its nanoscale dimensions and surface chemistry may influence the polarization of immune cells like macrophages, an area of emerging fundamental research [125,213]. Zhang et al. developed a sulfonated bacterial cellulose/chitosan hydrogel (MTX-SBC/CS Gel) for precision treatment of psoriasis [212]. By introducing sulfonic groups into bacterial cellulose, the material was endowed with potent reactive oxygen species (ROS)-scavenging capacity, while optimizing the drug compatibility and release kinetics of methotrexate (MTX). The incorporation of chitosan imparted self-healing and bioadhesive properties through hydrogen-bonding networks. In an imiquimod-induced psoriatic mouse model, this drug-loaded hydrogel exhibited therapeutic efficacy comparable to the clinical first-line drug betamethasone: significantly accelerating epidermal normalization and suppressing inflammatory cytokine expression. Notably, the sulfonated bacterial cellulose hydrogel alone (without MTX) also demonstrated therapeutic activity, suggesting intrinsic immunomodulatory functionality of the material itself. Transcriptomic analysis revealed the multimodal mechanism of sulfonated bacterial cellulose: downregulation of IL-23/Th17 axis components, inhibition of keratinocyte hyperproliferation, and restoration of redox homeostasis via HSP90α-mediated signaling pathways. This study integrates the drug delivery function of cellulosic materials with their intrinsic bioactivity, offering a novel strategy for the treatment of autoimmune skin diseases.

#### 3.4.3. Transdermal Drug Delivery Systems

For diseases requiring systemic treatment (e.g., chronic pain, hormone replacement therapy), cellulosic materials also demonstrate potential as components of transdermal drug delivery systems (TDDS), such as backing layers or drug reservoirs [214,215]. Chemical modification to regulate the hydrophilicity/hydrophobicity and microporous structure of cellulose membranes allows precise control over the rate of drug permeation from the patch into the skin [216]. Combining nanocellulose with chemical penetration enhancers (e.g., terpenes, fatty acids) can also enhance the transdermal delivery efficiency of macromolecular or hydrophilic drugs, offering new possibilities for the non-injectable administration of proteins and peptides [214].

### 3.5. Applications in Smart Wearable and Skin Monitoring

With the rapid advancement of flexible electronics and wearable technology, cellulose-based materials have emerged as fundamental platforms for constructing intelligent skin monitoring systems, owing to their excellent mechanical properties, biocompatibility, and potential for functional modification. By incorporating conductive components—such as conductive polymers, carbon-based materials, or metal nanoparticles—into cellulose networks, multifunctional hydrogels integrating sensing capabilities and therapeutic activity can be fabricated, enabling real-time monitoring of human motion, physiological signals, and wound microenvironment.

CNC, characterized by their high aspect ratio and abundant surface hydroxyl groups, exhibit unique advantages in constructing self-healing conductive hydrogels. Liu et al. developed a fast self-healing conductive hydrogel mediated by CNC, engineered through dynamic covalent bonds and controlled dual hydrogen bond crosslinking networks. This material demonstrated excellent self-healing properties and strain sensitivity, enabling real-time monitoring of physiological activities such as joint motion and swallowing [217]. Ullah et al. prepared CNC-reinforced hydrophobic association conductive hydrogels, achieving a fracture stress of 371.2 kPa, fracture strain of 2108%, exceptional self-healing efficiency, electrical conductivity of 420 mS/m, and a gauge factor (GF) of 7.4 at 750% strain. This material functions as electronic skin for monitoring joint movements of fingers, elbows, and neck [218]. Cong et al. constructed a glutaraldehyde-PVA/CNC/PEDOT:PSS conductive hydrogel through multiple hydrogen bonding systems. The material exhibited mechanical strength of 411 kPa, elongation of 580%, and self-healing efficiency exceeding 98.3%. As a strain sensor, it demonstrated a GF of 2.5 within the 0–580% strain range and, when integrated with 5G transmission modules, enabled remote data monitoring for self-powered electronic skin [219].

BC-based conductive hydrogels also show promising prospects in skin monitoring applications. Hu et al. developed a polyaniline-coated bacterial cellulose-reinforced PVA-based anisotropic conductive hydrogel (PTPB). Through directional freeze–thaw processing, the material formed anisotropic microstructures, achieving a fracture stress exceeding 2 MPa and toughness of 3.75 MJ/m^3^. It maintained stable sensing accuracy within a 200% strain range and could monitor human motion and electrocardiogram (ECG) signals in real time, with performance comparable to commercial Ag/AgCl electrodes [220]. Yan et al. developed a biomimetic multifunctional conductive hydrogel based on hydroxypropyl methylcellulose (HPMC) and tunicate cellulose nanocrystals (TCNCs). This material exhibited ultra-softness (modulus 10.04 kPa), ultra-high stretchability (4520%), and excellent fracture toughness (6.128 MJ/m^3^). It functioned as a strain sensor (GF = 7.57), pressure sensor (sensitivity 2.96 kPa^−1^), and temperature sensor (temperature coefficient of resistance −2.47%/°C), enabling real-time monitoring of human motion, writing trajectories, and temperature changes during photothermal therapy [221]. Chen et al. prepared polymer-grafted CNC-reinforced anti-swelling conductive hydrogels that maintained mechanical and sensing performance after 14 days of water immersion. These hydrogels could monitor human motion in both air and water, offering new strategies for underwater strain sensing applications [222]. Ji et al. developed waterborne polyurethane/bacterial cellulose (WPU/BC) composite flexible films. When integrated with flexible EGaIn electrodes, these films reliably detected human ECG signals, providing flexible and durable substrates for wearable device applications [223].

In the realm of intelligent wound microenvironment monitoring, Yuan et al. developed a smearable C-P-T/mCQDs hydrogel based on cellulose nanofibers (CNF). By embedding surface-carboxylated carbon quantum dots, this hydrogel exhibited sensitive pH-responsive color changes within the pH 4–9 range, enabling visual indication of wound infection and healing status. Simultaneously, the material demonstrated photodynamic antibacterial activity, achieving over 90% bactericidal rate against various bacteria within 20 min under 808 nm near-infrared irradiation, thereby integrating wound monitoring and therapy [224]. Zubova et al. loaded elderberry anthocyanins into bacterial cellulose to fabricate colorimetric smart hydrogel films. These films enabled pH evaluation via smartphone applications, with a detection limit of 3.45 A.U., providing a convenient tool for early diagnosis of bacterial infection [225].

## 4. Conclusions

Cellulose and its derivatives, owing to their distinct advantages of wide availability, excellent biocompatibility, and inherent potential for structural engineering modification, have become a research focus in the field of skin dressings. This review systematically summarizes the application progress of cellulose-based materials in skin dressings, encompassing various forms such as CMC, CNC, CNF, and BC, as well as diverse construction strategies including films, nanofibrous scaffolds, hydrogels, and aerogels. In the treatment of skin diseases, cellulose-based drug delivery systems establish an active defense system by combining physical barrier function with chemical antimicrobial action, provide biomimetic scaffolds for cell proliferation and tissue regeneration by simulating extracellular matrix structure, and dynamically regulate the wound microenvironment through their high hydrophilicity and porous structure, exhibiting a functional leap from “passive coverage” to “active intervention”. In recent years, the development of intelligent responsive cellulose-based gels has been particularly rapid. By introducing multiple stimuli-responsive mechanisms such as pH, temperature, enzymes, reactive oxygen species (ROS), glucose, and light, researchers have achieved precise sensing of complex wound microenvironments and on-demand drug release. Particularly for clinical challenges such as chronic diabetic wounds and drug-resistant bacterial infections, multi-responsive hydrogels based on chemical structures like dynamic Schiff base bonds, phenylboronate esters, and thioketals have demonstrated excellent on-demand therapeutic potential. Simultaneously, the application exploration of cellulose-based materials in fields such as sequential burn treatment, skin immunomodulation, pathological scar prevention, and transdermal drug delivery continues to expand. Although cellulose-based dressings have made significant progress in laboratory research (with the number of research publications increasing by nearly 50% from 2023 to 2025), their clinical translation still faces challenges including standardization of large-scale production, long-term in vivo biosafety evaluation, and insufficient design of intelligent responsiveness targeting dynamic pathological processes. Future research should focus on the synergistic integration of multiple responses, theranostics integration, and the development of AI-based intelligent feedback systems. Through interdisciplinary collaboration among materials science, bioengineering, and clinical medicine, the translation of cellulose-based dressing systems from the laboratory to clinical and daily-life applications can be promoted, providing more efficient, safer, and more personalized treatment options for patients with skin diseases worldwide. Finally, the main limitation of this review is that most of the cited studies are preclinical, with a lack of large-scale, randomized controlled human clinical trial data. Furthermore, the discussion on the long-term safety of cellulose-based dressings (such as the metabolic pathways of degradation products, risks of chronic inflammation or immunogenicity) and the issue of batch consistency in large-scale production remains insufficient.

## Figures and Tables

**Table 1:** Representative studies of cellulose-based smart responsive systems classified by triggering signals.

Type of Responsiveness	Carrier or Wall Materials	Cargo	Responsive Unit	Experimental Category	Effect	Reference
pH-Responsive	silane-modified bacterial nanocellulose	beet red pigment extract (BRPE)	beet red pigment extract	in vitro	Antioxidant, monitoring wound	[87]
	porous chitosan-based scaffolds, cellulose nanocrystals, graphene oxide	amino acid L-Arginine	-COOH, -NH2	in vitro	Anti-bacteria, proliferation and migration of cells, collagen deposition	[88]
	Medulla Tetrapanacis-lignocellulosic aerogel	Neohesperidin (Ne), silk fibroin (SF)	SF	in vitro, in vivo	Antioxidant, anti-inflammatory, proliferation and migration of fibroblasts, re-epithelialization, collagen deposition, delivery system	[89]
	chitosan/dialdehyde carboxymethyl cellulose	geniposidic acid	Schiff base	in vitro, in vivo	Anti-bacteria, re-epithelialization, angiogenesis	[90]
	carboxymethyl cellulose	citric acid, insulin	-COOH	in vivo	Delivery system	[91]
Temperature-responsive	poly(N-isopropylacrylamide) (PNIPAM)	water-soluble cellulose acetate (WSCA), ciprofloxacin	PNIPAM	in vitro, in vivo	Anti-bacteria, delivery system, hemostasis, re-epithelialization, collagen fiber remodeling, anti-inflammatory, reduced scar formation	[92]
	chitosan/hydroxyethyl cellulose/glycerophosphate	Cord blood mononuclear cells (CB-MNCs)	chitosan/glycerophosphate	in vivo	Anti-inflammatory, proliferation of cells, angiogenesis, collagen synthesis	[93]
	decanoic acid-modified chitosan (CSDA) and methyl cellulose (MC)	/	hydrophobic interactions, hydrogen bond and electrostatic attractions	in vitro, in vivo	Proliferation and migration of L929 cells and fibroblasts, angiogenesis, collagen deposition, re-epithelialization	[94]
Enzyme-Responsive	bacterial cellulose	thrombin	a recombinant thrombin-cellulose binding domain (CBD) fusion protein	in vitro, in vivo	hemostasis, angiogenesis	[95]
ROS-Responsive	a carboxymethyl cellulose-fabricated dissolvable microneedle (B/S-TM@MN)	berberine (Ber) and sinomenine (Sin)	thioketal bond (-S-C-S-)	in vivo	anti-inflammatory, anti-angiogenesis, delivery system	[96]
	cysteamine-modified cellulose nanospheres (Cys-Cel NS)	solid acid Amberlyst-15 (A15)	hydrogen bonds (-H…Y)	in vitro	antioxidation	[97]
Glucose-Responsive	cellulose nanofibers (CNFs)	gold nanoparticles (AuNPs), glucose oxidase (Gox)	Gox (FAD → FADH_2_)	in vivo	excellent conductivity and flexibility	[98]
Light-Responsive	norbornene modified carboxymethyl cellulose	indocyanine green (ICG), doxorubicin (DOX)	ICG	in vitro	delivery system	[99]
ROS, Light-Responsive	biomimetic hydroxyethyl cellulose (HEC)	Prussian blue (PB)	HEC, PB	in vitro	anti-bacteria	[100]
Temperature, Light-Responsive	sodium carboxymethyl cellulose (CMC), gelatin	polydopamine (PDA) calcium peroxide(CPO)-loaded polycaprolactone microspheres (CPO@PCL), vancomycin(Van)-loaded polycaprolactone microspheres (Van@PCL), and cerium oxide nanoparticles (CeNPs)	PDA, CPO@PCL, Van@PCL, CeNPs	in vitro, in vivo	Antioxidation, anti-inflammatory, anti-bacteria, trained immunity, metabolic reprogramming	[101]
	dopamine grafted oxidized pectin, carboxymethyl cellulose bearing hydrazide groups and hemostatic polyphosphate moiety	Polydopamine-coated graphene oxide (PGO), tannic acid (TA)	PGO, TA	in vitro, in vivo	Antioxidation, anti-inflammatory, tissue regeneration, anti-bacteria, collagen deposition, angiogenesis	[102]
pH, Temperature, Light-Responsive	cellulose nanocrystals grafted phenylboronic acid (CNCs-ABA), multiwalled carbon nanotubes (MWCNTs), polyvinyl alcohol (PVA)	NaOH	borate bonds (-B-O-), MWCNTs	in vitro	Conductivity enhanced, detecting human motion with superior biocompatibility and fast resistance response to applied strain	[103]
pH, ROS-Responsive	phenylboronic acid-grafted oxidized methylcellulose (POMC), poly(vinyl alcohol) (PVA)	type I recombinant human collagen (rhCOL1), mesoporous zinc oxide (mZnO)	boronate esters bonds (-B-O-)	in vitro, in vivo	Cell growth, angiogenesis, antibacterial, anti-inflammatory, skin regeneration	[104]
pH, Temperature Glucose-Responsive	phenylboronic-modified quaternized chitosan (QCS-PBA), polydopamine-coated tunicate cellulose crystals (PDAn@TCNCs) and polyvinyl alcohol (PVA)	insulin	Schiff base bonds (-C=N-) and phenylboronate esters (Ar–B(OR)_2_)	in vitro, in vivo	Anti-bacteria, delivery system, enhanced adhesion and antioxidation, hemostasis, angiogenesis	[105]

## Data Availability

No new data were created or analyzed in this study. Data sharing is not applicable to this article.
